# MRI-Based Prediction of Immunotherapy Response in Breast Cancer: A Systematic Review

**DOI:** 10.3390/cancers18203267

**Published:** 2026-10-10

**Authors:** Juhi Mehta, Cassidy Adams, Christian Sellers, Kevin E. Duong, Takouhie Maldjian

**Affiliations:** 1 Department of Radiology, Montefiore Medical Center, Albert Einstein College of Medicine, New York, NY 10461, USA; juhi.mehta@einsteinmed.edu (J.M.); cassidy.adams@einsteinmed.edu (C.A.); 2009kevinduong@gmail.com (K.E.D.); 2 School of Medicine, Meharry Medical College, Nashville, TN 37208, USA; christian.sellers@mmc.edu

**Keywords:** breast cancer, magnetic resonance imaging, immunotherapy, immune checkpoint inhibitors, neoadjuvant chemotherapy, neoadjuvant chemoimmunotherapy, pathologic complete response, diffusion-weighted imaging, radiomics, multiparametric imaging

## Abstract

**Background:** Immune checkpoint inhibitors (ICIs) have been shown to improve long term outcomes and pathological complete response (pCR) rates in triple-negative breast cancer (TNBC). However, treatment response remains heterogeneous. Magnetic resonance imaging (MRI) has emerged as a promising noninvasive biomarker for predicting response to immunotherapy-containing treatment regimens. **Purpose:** To systematically review studies evaluating MRI biomarkers for predicting response to immunotherapy-containing treatment regimens in breast cancer. **Methods:** A PRISMA-guided systematic review of PubMed was performed for studies published between June 2019 and June 2026 evaluating MRI-based prediction of pCR or immunotherapy response in breast cancer. Data regarding study design, MRI techniques, predictive performance, and study characteristics were extracted and qualitatively synthesized. **Results:** Across the ten included studies, conventional MRI generally demonstrated moderate predictive performance (AUC 0.65–0.75), whereas multiparametric radiomics models achieved the highest predictive performance (AUC up to 0.88). Several consistent themes emerged. Studies evaluating neoadjuvant chemoimmunotherapy (NACI) reported increased MRI-pathology discordance and reduced reliability of enhancement-based assessment alone, suggesting that conventional MRI response assessment may require refinement in this treatment setting. Functional MRI biomarkers, including diffusion-weighted imaging and dynamic enhancement metrics, identified treatment response earlier than baseline morphologic imaging, while radiomics models integrating MRI with clinical or molecular biomarkers consistently demonstrated the strongest predictive performance. However, evidence remained limited by heterogeneous imaging protocols, small study populations, and limited external validation. **Conclusions:** MRI, particularly multiparametric and radiomics-enhanced approaches, is evolving beyond conventional morphologic assessment to provide complementary information regarding tumor biology and treatment response in patients undergoing NACI. However, standardized imaging protocols and prospective multicenter validation are needed before MRI-derived biomarkers can be incorporated into routine clinical decision-making.

## Simple Summary

Immune checkpoint inhibitors improve outcomes for patients with triple-negative breast cancer, but not all patients respond equally to treatment. Identifying which patients are most likely to benefit remains an important challenge. Magnetic resonance imaging (MRI) offers a noninvasive way to evaluate tumors and may provide information beyond conventional imaging by reflecting features of tumor biology and early treatment response. This systematic review examines recent studies evaluating MRI for predicting response to immunotherapy-based treatment. We found that advanced MRI techniques, particularly functional imaging and radiomics, showed greater predictive ability than conventional MRI and may provide additional information about tumor biology and treatment response. However, current evidence is limited by small study populations and differences in imaging methods. Larger, standardized studies are needed before these MRI approaches can be routinely used to guide treatment decisions in clinical practice.

## 1. Introduction

Breast cancer is the second most common cancer worldwide, and is the most frequently diagnosed cancer in women globally, accounting for 11.6% of cancers globally in 2022 [1,2]. Triple-negative breast cancer (TNBC) is an immunohistochemical subtype of breast cancer defined by its lack of expression of estrogen receptor (ER), progesterone receptor (PR), and human epidermal growth factor receptor-2 (HER-2) receptor. It is characterized by worse prognosis, more aggressive behavior, and shorter survival compared with other breast cancer subtypes [3]. Neoadjuvant chemotherapy (NAC) alone has long been the cornerstone of treatment for early-stage triple-negative breast cancer (TNBC) [4]. The advent of immune checkpoint inhibitors (ICIs) has been shown to improved long-term outcomes and has transformed the treatment landscape for eligible patients [4,5]. ICIs function by binding to and blocking inhibitory checkpoint proteins, most notably programmed cell death protein 1 (PD-1) and programmed cell death-ligand 1 (PD-L1), thereby reactivating cytotoxic T-cell-mediated antitumor immunity [6]. Pembrolizumab is now established as a standard-of-care component of neoadjuvant chemoimmunotherapy (NACI) in high-risk, early-stage TNBC based on the KEYNOTE-522 trial, with additional evidence supporting atezolizumab and durvalumab in this setting [7,8,9,10].

Pathologic complete response (pCR), defined as the absence of residual invasive carcinoma in both the breast and axillary lymph nodes at definitive surgery, has emerged as a critical endpoint in the neoadjuvant paradigm [11,12]. This is assessed at the time of definitive surgery and may be defined according to specific criteria such as ypT0/TisypN0. In this review, immunotherapy response refers broadly to the response of breast cancer to an immunotherapy-containing neoadjuvant treatment regimen. Treatment response is a broader term describing the response of the tumor to neoadjuvant therapy and may be assessed using imaging or pathological measures. In the present review, MRI findings were evaluated as potential predictors of these treatment-response outcomes, with the specific outcome definition determined according to the individual study.

Achieving pCR is strongly associated with improved event-free and overall survival, with the prognostic significance being most pronounced in aggressive subtypes such as TNBC [12,13]. Consequently, pCR has been endorsed by regulatory agencies as an endpoint for accelerated drug approval in the neoadjuvant breast cancer setting [12]. The addition of ICIs to NAC has substantially improved pCR rates in TNBC, as demonstrated by multiple randomized trials [6,8,9]. Despite these advances, a substantial proportion of patients fail to achieve pCR, underscoring the need for reliable, non-invasive biomarkers capable of predicting treatment response early in the therapeutic course [14,15].

Magnetic resonance imaging (MRI) is the most accurate imaging modality for evaluating breast cancer treatment response, surpassing mammography and ultrasound [16,17]. In the neoadjuvant setting, MRI provides both anatomical and functional assessment through complementary sequences: dynamic contrast-enhanced MRI (DCE-MRI) captures tumor vascularity and perfusion kinetics (which include Ktrans, Ve, and Kep, and which may have different biological implications), diffusion-weighted imaging (DWI) with apparent diffusion coefficient (ADC) mapping reflects tissue cellularity, and T2-weighted sequences provide morphological information [18,19,20]. Radiologic complete response (rCR), defined as the absence of measurable tumors and abnormal features in lymph nodes as evaluated by medical imaging, on MRI has shown strong concordance with pCR, particularly in TNBC treated with NACI [21]. The integration of advanced computational approaches, including radiomics, habitat imaging, and machine learning, with multiparametric MRI data has shown considerable promise in enhancing predictive performance for pCR beyond conventional imaging assessment [18,22,23]. Importantly, longitudinal MRI assessment at multiple treatment timepoints has been shown to improve predictive accuracy compared with single-timepoint evaluation, as early changes in ADC and tumor morphology can precede volumetric response and provide actionable information for treatment adaptation [19,24,25]. In the specific context of chemoimmunotherapy, recent multicenter studies have demonstrated that post-NACI treatment MRI combined with clinical variables can yield high discriminative performance for predicting pCR in TNBC [26].

However, the role of MRI in the immunotherapy era remains controversial and incompletely understood. Emerging evidence suggests that immunotherapy may fundamentally alter the relationship between radiologic and pathologic findings; immune-mediated inflammatory changes in the tumor bed can mimic residual disease on MRI, leading to reduced concordance between rCR and pCR and a significantly diminished negative predictive value of post-treatment MRI in patients receiving NACI compared with NAC alone [27]. This discordance raises critical questions about whether conventional MRI interpretation criteria, developed and validated in the chemotherapy-only era, remain reliable when ICIs are incorporated into neoadjuvant regimens. Furthermore, while several reviews have examined MRI-based radiomics for predicting response to NAC broadly, and individual studies have begun to explore MRI performance specifically in the NACI setting, no comprehensive systematic review has synthesized the evidence on MRI-based prediction of pCR in breast cancer patients receiving NACI [26,28,29,30,31,32]. The existing literature is fragmented across heterogeneous study designs, imaging protocols, MRI sequence types, analytical methodologies, and treatment timepoints, making it difficult for clinicians and researchers to draw unified conclusions.

This systematic review addresses this critical gap by comprehensively evaluating the current evidence on MRI for predicting pCR in breast cancer patients receiving NACI, synthesizing findings on imaging modalities, predictive performance, and the potential role of advanced predictive models in guiding treatment personalization in the immunotherapy era.

## 2. Materials and Methods

No Ethics committee approval was required for this systematic review.

### 2.1. Eligibility Criteria

Studies were eligible for inclusion if they met the following criteria: (1) included patients with breast cancer receiving NACI, (2) included an MRI performed pre-treatment or early during therapy, and (3) Reported pCR outcomes/predictive metrics (AUC, sensitivity, specificity, percent achieving pCR, false negative rate, false positive rate). Studies were not eligible for inclusion if they met the following criteria: (1) They were case reports, reviews, or non-human studies, (2) Studies without MRI or immunotherapy data. The data extracted from eligible studies included country/institution, number of patients, tumor type, MRI image types, ground truth (pCR), predictive metrics, and study notes. No minimum sample size was specified as an inclusion or exclusion criteria, and studies were not excluded based on sample size. No specific MRI acquisition or interpretation protocol was required for eligibility.

Given the small number of included studies (*n* = 10) and substantial clinical, methodological, and imaging heterogeneity across studies, quantitative meta-analysis was not performed, as pooling estimates was considered unlikely to yield a meaningful summary effect and could potentially result in misleading conclusions.

### 2.2. Search Strategy

This systematic review was conducted in accordance with the PRISMA 2020 (Preferred Reporting Items for Systematic Reviews and Meta-Analyses) guidelines [33] and has not been registered. Given the focused and exploratory nature of the review and the evolving terminology used in this emerging field, a prospective registry protocol was not established before the literature search. We acknowledge this as a limitation. The literature search included all articles published from 14 June 2019 to 14 June 2026. The start date was selected to capture emerging evidence on MRI-based response to neoadjuvant immunotherapy during the period in which immunotherapy was becoming an increasingly relevant component of neoadjuvant treatment of high-risk triple-negative breast cancer.

A systematic literature search was conducted in PubMed using predefined combinations of keywords and Boolean operators (AND).

- Search 1: “breast cancer” AND “MRI” AND “immunotherapy” AND “pCR”—15 records retrieved; 7 were excluded as unrelated.
- Search 2: “breast cancer” AND “MRI” AND “tumor response”—78 records retrieved; 76 were excluded as unrelated and 1 was identified as a duplicate from the first search, leaving 1 additional eligible study (Spriet et al.).
- Search 3: “breast cancer” AND “MRI” AND “immunotherapy response”—5 records retrieved; 1 additional eligible study was included (Han et al.).

### 2.3. Study Selection and Data Extraction

Study selection and eligibility screening were independently performed by JM and CA and verified by TM and/or KED. No discrepancies were identified during the data extraction process, therefore adjudication by a third reviewer was not required. Only original research articles published in English were included.

**Figure 1:**
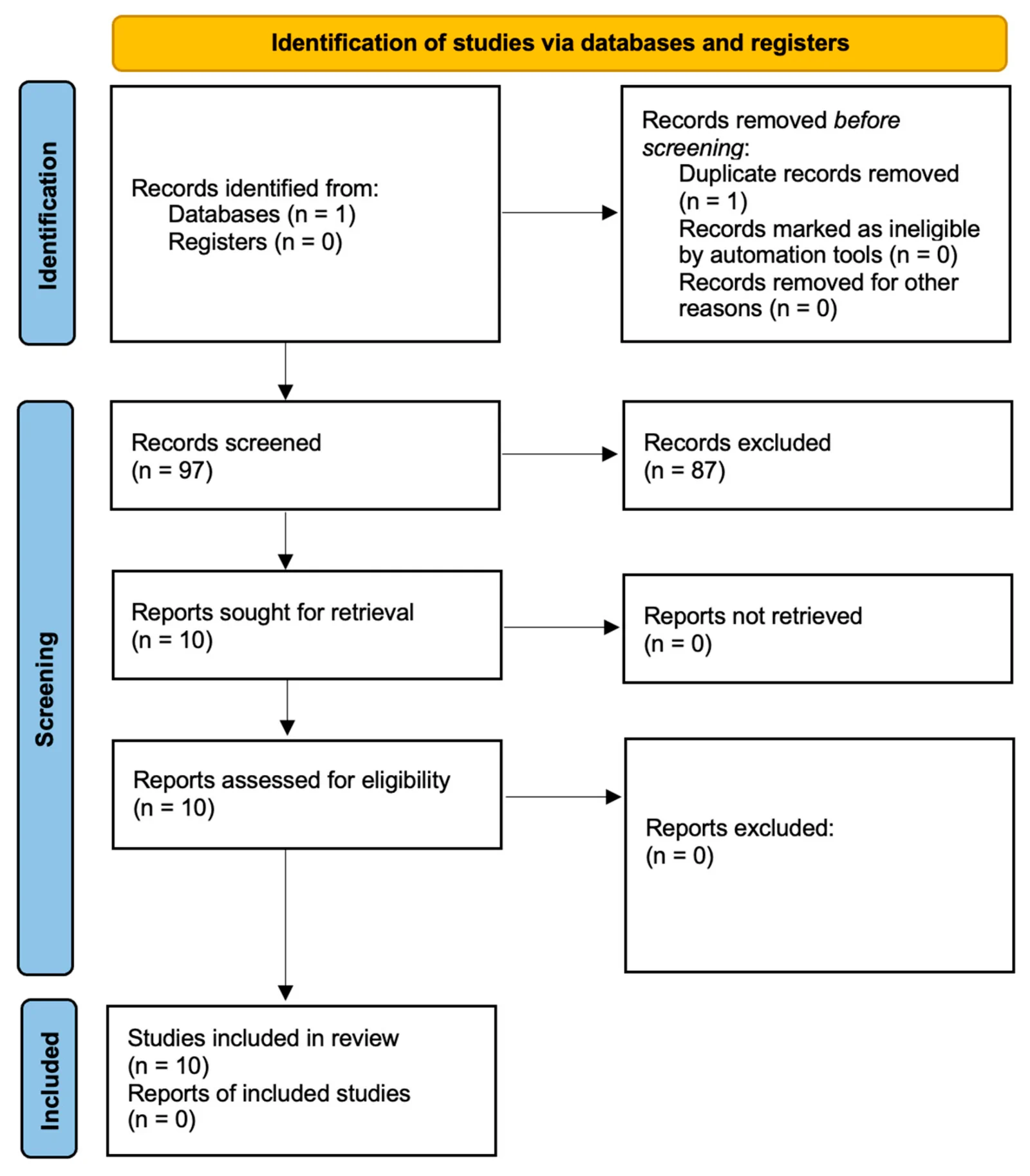
PRISMA 2020 flow diagram of study identification, screening, and inclusion [33].

A PRISMA flow diagram was used to track study selection (Figure 1).

## 3. Results

Across the included studies, MRI demonstrated moderate to high performance for predicting response to NACI in breast cancer, particularly in TNBC (Table 1). Although methodologies varied substantially, several consistent themes emerged regarding the value of multiparametric imaging (combination of MRI pulse sequences e.g., DCE-MRI, DWI, T2-weighted), diffusion-weighted techniques, radiomics, and early treatment-response assessment. While functional MRI techniques such as DCE-MRI and DWI/ADC provide quantitative metrics that may correlate with early response [24,31], radiomics and AI-driven analyses capture complex patterns beyond human interpretation [32,34].

### 3.1. Conventional MRI and Radiologic Complete Response

Several studies evaluated the ability of DCE- MRI to predict pCR after NACI. Several studies reported favorable MRI performance in NACI-treated cohorts, although methodologies varied and direct comparisons with NAC-treated cohorts were limited. Spriet et al. (2025) [35] prospectively evaluated abbreviated ultrafast MRI (UF-MRI), a high-temporal-resolution DCE-MRI technique that rapidly acquires sequential images during early post contrast period. In this study, UF-MRI consisted of a Rapid Twist-Vibe Dixon T1-weighted 3D gradient-echo sequence with 10 postcontrast acquisitions obtained in 8-s intervals. Compared with conventional DCE-MRI, UF-MRI provides more frequent temporal sampling of the initial contrast wash-in, and therefore emphasizes early enhancement kinetics related to tumor vascularity and perfusion, whereas conventional DCE-MRI typically provides higher spatial resolution and morphologic characterization across later postcontrast phases. UF-MRI was compared with full protocol MRI for predicting tumor regression/pCR in 36 patients with early TNBC receiving NAC plus pembrolizumab. Images were interpreted collaboratively by 2 radiologists. Both protocols demonstrated comparable ability to predict pCR, with sensitivities of 0.83 and 0.78, respectively for UF-MRI versus the full-protocol, and identical specificities of 0.69. Notably, UF-MRI correctly identified one case falsely interpreted as residual disease on full-protocol MRI, likely because delayed enhancement related to treatment-associated inflammation or fibrosis was less pronounced on UF-MRI [35]. Overestimation of persistent tumor due to reactive inflammation, fibrosis or necrosis following treatment with NAC can result in late enhancement and overestimation of residual tumor [40,41,42], and was offered as an explanation for the false negative case with the full-protocol MRI that was correctly evaluated by UF-MRI. The study suggests that pembrolizumab-induced inflammatory changes may complicate interpretation of conventional delayed enhancement patterns. Importantly, UF-MRI reduced acquisition time by approximately 9 min without compromising diagnostic accuracy, supporting its potential role in routine response monitoring. Decreased volume of images may also reduce interpretation time. One patient achieved pCR with one dose of pembrolizumab; raising the possibility that some patients may not require the full dose of pembrolizumab treatment and could avoid unnecessary side effects while still achieving effective treatment outcomes, a hypothesis that warrants further investigation. Weaknesses of the study include a small sample size (*n* = 36) and a single center study design, which may limit the generalizability of its findings. Additionally, analysis of UF-MRI consisted of a visual analysis, rather than a precise analysis of kinetic parameters of enhancement, warranting further investigation into optimal abbreviated MRI protocols.

Similarly, Damião et al. (2026) [21] compared MRI accuracy in predicting pCR with NAC alone in a cohort of 59 TNBC patients versus NACI (NAC plus pembrolizumab) in a cohort of 52 patients between 2021 and 2024 in a retrospective single-center study. All patients underwent MRI before treatment and again prior to surgery. Images were interpreted by two radiologists with discordant cases resolved by consensus. Patients receiving NACI demonstrated significantly higher rates of both preoperative rCR (75% vs. 44.1%, *p* < 0.001) and pCR (67.3% vs. 47.5%, *p* = 0.025). Importantly, MRI also showed improved diagnostic performance in predicting pCR in patients treated with NACI as compared to NAC, with higher sensitivity (94.3% vs. 67.9%), higher overall accuracy (84.6% vs. 72.8%), and AUC improvement from 0.602 to 0.736 (though not statistically significant, possibly due to small cohorts). These findings demonstrate significantly higher rCR and pCR rates among patients receiving NACI, while MRI also showed numerically improved diagnostic performance compared with NAC alone. However, the difference in AUC was not statistically significant, and the small cohort sizes warrant cautious interpretation of the comparative imaging findings. The data supports the hypothesis that immunotherapy may modulate tumor biology in a manner that enhances the detectability of treatment response by medical imaging. However, despite improved sensitivity, MRI interpretation remained vulnerable to false-positive findings related to treatment-induced enhancement abnormalities, similar to those discussed in the above paragraph (inflammation, fibrosis, or necrosis) that can mimic residual disease on post-treatment imaging [21]. Limitations of this study include its retrospective, single-center design and a reader-dependent definition of rCR.

Javed et al. (2026) [39] also observed persistent discordance between imaging and pathologic response by subtype in a retrospective single center study of 201 early breast cancer patients receiving neoadjuvant systemic therapy over a 5-year period. Among TNBC patients, a cohort of 27 received NAC plus pembrolizumab, while 37 received NAC. Radiology MRI reports were used to assess radiologic response, with final surgical pathology serving as the reference standard. As per established protocols, no TNBC patients with T1N0 disease received NACI, which constricted the cohorts to 27 TNBC patients who received NACI + NAC and 29 TNBC patients who received only NAC. Among patients with TNBC, pCR was numerically higher in the NAC plus pembrolizumab group (14/27) than the NAC-alone group (13/29) after exclusion of T1 tumors (<2 cm) (51.9% vs. 45%). pCR rates were highest in HER2-positive (60.2%) and TNBC (53.1%) tumors, and lowest in ER-positive/HER2-negative disease (11.1%). Among HER2-positive cases, ER-negative tumors showed higher pCR rates than ER-positive tumors (75.8% vs. 50.0%). Within the TNBC subgroup, MRI demonstrated a false-negative rate of 15.3% for predicting pCR. However, MRI diagnostic performance was not reported separately for patients receiving NAC plus pembrolizumab (*n* = 27) and those receiving NAC alone (*n* = 37), limiting interpretation of immunotherapy-specific imaging findings. MRI demonstrated a low false-positive rate of 1.1%, with performance varying across subtypes. Overall, the findings suggest that although pCR rates in more aggressive subtypes align with those seen in clinical trials, MRI limitations, particularly its tendency to overcall residual disease, pose challenges for guiding surgical de-escalation [39]. Interpretation was limited by the small sample size and the retrospective, single-center design. Additionally, imaging assessments were based on routine clinical reports rather than a central review performed by independent readers.

Collectively, these studies reinforce the concern that post-immunotherapy inflammatory or fibrotic enhancement may mimic viable tumor and limit the reliability of morphology-based assessment alone. The study by Spriet et al. suggests that utilizing UF-MRI protocol may mitigate the tendency of MRI to overcall residual disease [35].

### 3.2. MRI Response Patterns in the Immunotherapy Era

Multiple studies demonstrated that tumor response patterns after NACI differ from those historically observed with NAC alone, suggesting that conventional MRI response criteria may not fully apply in the setting of immunotherapy.

Zou et al. (2024) [37] specifically compared MRI shrinkage patterns in TNBC patients treated with NAC alone (*n* = 65) versus NACI (*n* = 34). The immunotherapy patients received was defined as either a non-anthracycline or an anthracycline-based regimen of four cycles of PD-1 inhibitor, but a specific immunotherapy drug was not specified. They had 2 breast radiologists categorize first-post treatment MRI after first immunotherapy cycle compared to pretreatment MRI as concentric shrinkage, diffuse shrinkage, or no change, evaluating based on DWI and fat suppressed T2-weighted MRI (T2WI). Diffuse decrease pattern of tumor shrinkage was substantially more common in the NACI group (68% vs. 36%), whereas concentric shrinkage predominated in NAC-only patients (53% vs. 20%). Despite these atypical imaging appearances, pCR rates remained high across all shrinkage patterns in the NACI cohort. These findings suggest that NACI alters tumor regression kinetics and spatial response patterns, potentially reducing the reliability of traditional size-based imaging criteria. The pCR rate was higher for the NACI cohort, at 82% for the NACI cohort and 45% for the NAC cohort. 56% of NACI patients with diffuse decreased pattern (the more common pattern in NACI cohort seen in 68% of NACI patients) achieved pCR, while only 17% of the concentric shrinkage pattern (the less common pattern seen in the NACI cohort, seen in 20% of the NACI group) achieved pCR in the NACI cohort [37]. Limitations included a small sample size, and a retrospective study design.

Collectively, the findings from Spriet, Zou, Damião, and Javed indicate that immunotherapy changes tumor vascularity, immune-cell infiltration, and stromal composition in ways that may decouple MRI enhancement patterns from true residual tumor burden. As a result, persistent enhancement after immunotherapy may not necessarily represent viable disease [21,35,37,39].

### 3.3. Early Post-Treatment Changes in Diffusion-Weighted Imaging and Functional Biomarkers

Several studies suggested that DWI and ADC measurements may provide more biologically relevant response biomarkers than conventional DCE-MRI metrics during immunotherapy.

Li et al. (2022) [31] evaluated ADC-derived metrics from DWI compared with functional tumor volume (FTV) from DCE-MRI using a logistic regression model in HER2-negative patients with locally advanced disease enrolled in the I-SPY2 trial, a multicenter public database. ADC changes generally outperformed FTV for predicting pCR in patients receiving NAC plus pembrolizumab. Two readers were trained, then blinded to pathologic outcome, and assessed DWI independently. Discrepancies were resolved by a breast imager with ten years of experience. In the pembrolizumab cohort, the best-performing ADC metric achieved an AUC of 0.75 compared with 0.61 for FTV, whereas performance differences were less pronounced in chemotherapy-only controls. Mean ADC in the pembrolizumab cohort achieved an AUC of 0.73, compared to 0.63 in the NAC only (control) cohort. These findings suggest that diffusion-based measures of tumor cellularity may better reflect early biologic response to immunotherapy than vascular enhancement metrics, which may be confounded by immune-cell infiltration and treatment-related inflammation [31].

However, the study also highlighted important technical limitations of DWI. Nearly half of initially eligible patients were excluded (114 out of 249) because of inadequate DWI quality on pretreatment or 3 weeks post treatment MRI, emphasizing the need for standardized acquisition protocols and quality-control measures before widespread clinical implementation. Of the 132 patients included, 75 were treated with NAC only while 28 were treated with NAC plus pembrolizumab, demonstrating the limitation of sample size for the study group [31].

Liu et al. (2025) [36] further emphasized the importance of early functional imaging biomarkers. Using DCE-MRI in TNBC patients receiving NACI (NAC plus an unspecified PD-1 inhibitor), the investigators identified percentage enhancement reduction and early tumor shrinkage after only one treatment, which were compared to pretreatment tumor unifocality on MRI as independent predictors for pCR. Independent assessment was performed by 2 experienced MRI readers, followed by consensus discussion for pretreatment baseline MRI and first cycle NACI MRI. Shapley additive explanation analysis demonstrated that percentage enhancement reduction was the most important independent predictive feature, followed closely by early tumor shrinkage (greater than or equal to 37%), while tumor unifocality was least predictive. A predictive model constructed using multivariable logistic regression incorporating all three parameters achieved an AUC of 0.88, sensitivity of 74%, and specificity of 90% in the external test set. These findings support the concept that dynamic treatment-response kinetics may be more informative than baseline imaging characteristics alone. Strong concordance demonstrated between pCR outcomes and their MRI-based predictive model using all three parameters studied suggests potential utility for early response prediction. While such prediction could ultimately inform treatment de-escalation for responders or therapeutic adjustments for non-responders, the study did not evaluate MRI-guided treatment modification or whether such strategies improve patient outcomes [36]. Limitations include a small external validation sample size, a retrospective design, and a lack of long-term outcome data to validate the model’s abilities.

### 3.4. Radiomics, Artificial Intelligence, and Tumor Microenvironment Characterization on Pre-Treatment MRI

The highest predictive performance across studies was generally achieved using multiparametric radiomics and machine learning approaches, which aimed to capture intratumoral heterogeneity and features of the tumor microenvironment (TME).

Ramtohul et al. (2024) [32] prospectively evaluated pretreatment hand-crafted radiomics derived from pharmacokinetic DCE-MRI parameters in early-stage TNBC treated with NAC plus pembrolizumab. A radiomics score generated from K_trans_ (rate constant for contrast agent plasma-to-interstitial transfer), V_e_ (volume fraction of extravascular and extracellular space), and Slope_max_ (maximum contrast uptake rates) maps using LASSO technique achieved an AUC of 0.80 independently and 0.86 when combined with tumor grade and Ki-67. Importantly, higher radiomics scores correlated with tumor size, washout enhancement, PD-L1 positivity and androgen receptor expression, suggesting a link between MRI-derived pretreatment pharmacokinetic parameters and TME and underlying tumor biology. MRI-derived pharmacokinetic radiomic features on pretreatment DCE-MRI effectively predicted treatment response in TNBC patients, supporting a potential role for pretreatment MRI in tailored therapeutic decision-making [32]. Although externally validated, the model was developed specifically in patients treated with the KEYNOTE-522 regimen (NAC plus pembrolizumab), and its generalizability to other treatment regimens remains uncertain. Additionally, these pharmacokinetic parameters require quantitative modeling of DCE-MRI data, which may not be routinely available in clinical practice and could limit broader implementation of this approach.

In a subsequent prospective multicenter study, Ramtohul et al. (2025) [26] evaluated post-NACI treatment (NAC plus pembrolizumab) MRI of early TNBC patients using a hand-crafted radiomics model and demonstrated that rCR alone achieved an AUC of 0.83, improving to 0.88 when combined with nodal status and Ki-67 using an externally validated multivariable logistic regression model. Furthermore, among patients without rCR, additional radiomics analysis achieved an AUC of 0.80, suggesting that radiomics may provide complementary information even when residual enhancement persists [26]. However, several limitations should be considered. Though externally validated, the subgroup proposed for potential surgical omission was relatively small, requiring additional validation before clinical implementation. Furthermore, MRI acquisition across different scanner models may limit reproducibility despite protocol standardization.

Han et al. [34] extended this concept by integrating MRI radiomics with transcriptomic analysis of the TME. Using RNA-sequencing–derived immune phenotypes as the reference standard, the investigators identified immune-inflamed and immune desert tumor subtypes and developed a hand-crafted radiomics approach on DCE-MRI images with semi-automated segmentation where extracted features were subsequently analyzed using a Random Forest machine learning model capable of differentiating these phenotypes with AUCs ranging from 0.814 to 0.855 across cohorts. In a separate external validation cohort of 77 patients treated with anti-PD-1/PD-L1 therapy, higher baseline radiomics scores predicted objective response (complete or partial response according to RECIST 1.1) with an AUC of 0.784, suggesting that MRI-derived radiomic features may serve as a noninvasive surrogate of the underlying immune microenvironment and help identify patients most likely to benefit from immunotherapy [34]. Limitations of the study include its retrospective design and heterogeneous, multi-cohort dataset assembled from both public databases and a single institutional cohort. Additionally, the immunotherapy validation cohort included multiple breast cancer subtypes treated with anti-PD-1/PD-L1 therapy rather than exclusively patients with TNBC receiving neoadjuvant chemoimmunotherapy, which may limit direct applicability to the population of this review. Furthermore, the integration of MRI radiomics with transcriptomic analysis may limit the routine clinical implementation of this approach, as transcriptomic profiling is not routinely available in clinical practice.

The higher immune cell infiltration was also associated with significantly improved overall survival. These findings support the emerging role of MRI as a noninvasive surrogate biomarker of the TME [26,32,34].

### 3.5. Pretreatment Quantitative Habitat and Volumetric Analysis

Weinfurtner et al. explored quantitative volumetric habitat concentration analysis of pretreatment MRI in patients with TNBC, in which tumors were divided into imaging-defined ‘habitats’ based on voxel-level enhancement characteristics and the relative proportion of each habitat within the tumor was quantified [38]. The study included 25 TNBC patients at a single institution receiving intratumoral talimogene laherparepvec (T-VEC) immunotherapy combined with NAC. Patients achieving pCR demonstrated significantly higher baseline percent FTV and high-enhancement volume (HEV) habitat concentrations than nonresponders. Tumors with above-average pre-treatment enhancement habitat metrics (FTV or HEV), with greater concentration of enhancing higher intensity voxels showed markedly higher pCR rates, suggesting that pretreatment vascular and functional heterogeneity may influence immunotherapy sensitivity [38]. It should be noted however that the study included a small sample size and single-center pilot design, which may limit the generalizability of the findings. In addition, only patients receiving T-VEC combined with a specific neoadjuvant chemotherapy regimen were evaluated, so it is unclear whether these findings apply to other immunotherapy regimens. Finally, the habitat analysis required semi-automated segmentation and specialized software, which may limit broader clinical implementation.

### 3.6. Overall Predictive Trends

Taken together, the reviewed studies demonstrated a progression from conventional morphologic MRI assessment toward biologically driven imaging biomarkers. Conventional MRI achieved moderate predictive performance, particularly when assessing rCR after treatment. However, functional imaging approaches—including DWI/ADC, ultrafast imaging, and dynamic enhancement kinetics—show improved ability to characterize early treatment response [31,32,35]. Overall, multiparametric radiomics and AI-based models consistently demonstrated strong predictive performance, particularly when imaging features were integrated with clinical or molecular biomarkers such as Ki-67 and PD-L1 expression [26,32,34].

## 4. Discussion

This review demonstrates that MRI is emerging as a promising noninvasive biomarker for predicting pathologic complete response following neoadjuvant chemoimmunotherapy in breast cancer, particularly TNBC, with the included studies demonstrating potential across conventional MRI, functional imaging, and radiomics-based approaches. Across studies, MRI-based assessment evolved beyond conventional morphologic evaluation toward functional, quantitative, and AI-driven approaches capable of characterizing tumor biology and the immune microenvironment. However, despite this encouraging trajectory, the current body of evidence remains largely exploratory. Most studies evaluated surrogate endpoints such as pCR rather than long-term oncologic outcomes, and therefore it remains uncertain whether MRI-derived biomarkers translate into clinically meaningful improvements in event-free or overall survival. Consequently, the existing literature supports MRI as a promising investigational biomarker rather than an established clinical decision-making tool.

A major recurring theme was that tumor response patterns observed during NACI differ substantially from those historically described in the NAC-only era. Conventional MRI response criteria were largely developed based on NAC, where treatment response typically manifests as concentric tumor shrinkage and decreasing enhancement. However, studies by Zou et al., Spriet et al., Damião et al., and Javed et al. collectively demonstrated that immunotherapy alters these imaging patterns. Diffuse shrinkage, persistent enhancement, and MRI-pathology discordance were more frequently observed after NACI, likely reflecting treatment-induced immune-cell infiltration, fibrosis, necrosis, and vascular remodeling rather than viable residual tumor [21,35,37,39]. Consequently, enhancement persistence after immunotherapy may not reliably indicate treatment failure. Spriet et al. further demonstrated that an UF-MRI protocol may reduce MRI overestimation of residual disease while shortening acquisition time and potentially reducing interpretation time [35]. Collectively, these studies suggest that conventional MRI response assessment, which relies heavily on residual enhancement and tumor size reduction, may require modification for patients receiving NACI because treatment-induced inflammatory changes can mimic residual disease. Importantly, although these studies consistently reported altered imaging patterns, most involved relatively small cohorts and lacked standardized imaging-response criteria, limiting confidence in the reproducibility of these observations. Moreover, MRI-pathology discordance was generally interpreted as evidence of immune-related inflammation; however, histopathological correlation was often limited, making this explanation biologically plausible but not definitively established. Future studies incorporating comprehensive imaging-pathology correlation are needed to validate these proposed mechanisms.

These findings highlight an important conceptual shift: MRI in the immunotherapy era may be measuring tumor biology and immune activity rather than tumor size alone. This interpretation is supported by studies linking MRI features to the tumor immune microenvironment. Han et al. demonstrated that radiomic signatures derived from DCE-MRI could differentiate immune-inflamed from immune-desert phenotypes and predict response to PD-1/PD-L1 blockade [34]. Similarly, Ramtohul et al. identified associations between radiomics scores and PD-L1 positivity, suggesting that MRI may function as a surrogate marker of immune activation and tumor immunogenicity [32]. Broader radiogenomic research further supports an association between MRI-derived features and underlying breast tumor biology. Acquaah-Mensah et al. evaluated MRI radiomics alongside gene-expression data for prediction of molecular subtype and disease recurrence [43], while Li et al. developed a radiogenomic model linking breast MRI features with components of the tumor microenvironment, including cancer-associated fibroblasts and T-cell infiltration [44]. These studies support further investigation into whether integrating MRI with transcriptomic and tumor microenvironment data may improve treatment-response prediction.

Critically, however, these findings should not be interpreted to mean that MRI directly measures immune activity. Rather, radiomic signatures are indirect imaging surrogates influenced by multiple biological processes, and their relationship with immune-cell infiltration requires further validation using integrated imaging, histopathology, transcriptomics, and spatial immune profiling. Nevertheless, these studies suggest that MRI may offer a more comprehensive, noninvasive assessment of the tumor microenvironment than is possible with single-site tissue sampling alone. Accordingly, MRI-derived biomarkers may ultimately complement established clinical and molecular biomarkers and warrant further investigation for pretreatment patient stratification.

Another important trend was the increasing value of functional imaging biomarkers compared with conventional enhancement-based assessment. DCE-MRI primarily reflects tumor vascularity, permeability, and perfusion, all of which can be altered by treatment-related inflammatory changes. In contrast, DWI assesses tumor cellularity through ADC measurements and may therefore be less susceptible to confounding enhancement changes. Li et al. demonstrated that ADC metrics outperformed FTV measurements for predicting pCR in patients receiving pembrolizumab-containing therapy, supporting the hypothesis that DWI may better capture biologically meaningful treatment response during immunotherapy [31]. At the same time, the technical limitations of DWI remain significant. Nearly half of potentially eligible patients in the Li et al. study (114 of 249) were excluded because of inadequate DWI quality. This high exclusion rate may introduce selection bias and limit the generalizability of the reported DWI findings, as the predictive performance was derived only from patients with DWI of sufficient quality of analysis. It also highlights an important disconnect between research performance and real-world clinical feasibility. Biomarkers that perform well only under optimal imaging conditions may have substantially lower utility in routine practice, where image quality varies considerably across scanners and institutions. Accordingly, future studies should emphasize standardized acquisition protocols and reproducible imaging workflows in addition to predictive accuracy.

Another consistent finding across studies was the importance of dynamic and early-treatment imaging biomarkers. Static baseline imaging features alone generally showed lower predictive performance than early changes observed during therapy. Li et al. found that ADC changes at 3 weeks were predictive of pCR, while Liu et al. demonstrated that enhancement reduction and early tumor shrinkage after only one treatment cycle were highly predictive [31,36]. Spriet et al. further suggested that ultrafast early enhancement imaging may better distinguish viable tumor from delayed inflammatory enhancement [35]. Together, these findings indicate that treatment-response kinetics may be more informative than baseline tumor appearance, highlighting the value of longitudinal MRI assessment for evaluation response as therapy progresses. These evolving applications of MRI also raise the possibility that treatment response could be identified earlier in the neoadjuvant course, potentially allowing future studies to investigate MRI-guided treatment adaptation for responders and nonresponders. Despite these promising findings, the optimal timing for response assessment remains uncertain, as individual studies evaluated different imaging intervals and treatment milestones. This heterogeneity limits direct comparison between studies and suggests that standardized imaging time points will be necessary before early MRI response assessment can be incorporated into adaptive treatment algorithms.

The highest predictive performance was consistently achieved with multiparametric radiomics and machine learning models. Studies by Ramtohul et al. and Han et al. demonstrated AUCs approaching or exceeding 0.85 when combining radiomic features with clinical or molecular biomarkers. These approaches likely outperform conventional imaging because they capture complex intratumoral heterogeneity and subtle spatial-temporal patterns beyond human visual interpretation. Importantly, several radiomics models also demonstrated external validation across independent cohorts, supporting their potential generalizability [26,34]. However, external validation was generally limited in scale and often performed using cohorts with similar patient populations and imaging protocols. Therefore, true generalizability across different healthcare systems, MRI vendors, acquisition parameters, and ethnically diverse populations remains largely unproven. Furthermore, many machine learning models function as “non-interpretable models,” limiting clinician interpretability and potentially hindering clinical adoption despite excellent predictive performance. Explainable AI approaches that provide biologically meaningful feature interpretation may therefore be equally important as further improvements in predictive accuracy. For example, Liu et al. used Shapley additive explanation (SHAP) analysis to quantify the relative contribution of individual imaging features to model predictions, identifying percentage enhancement reduction as the most influential predictor, followed by early tumor shrinkage and tumor unifocality. Such approaches may improve model interpretability by allowing clinicians to understand which imaging features contribute most strongly to a prediction, rather than relying solely on overall measures of predictive performance.

The increasing use of machine-learning approaches in MRI-based prediction introduces both opportunities and methodological challenges. Explainable artificial intelligence (XAI) should not be viewed solely as an alternative to “black-box” models. In the context of predictive modeling, explainability can help identify which imaging features contribute to model predictions, facilitate assessment of biological or clinical plausibility, and assist in identifying potential sources of bias or instability. This is particularly relevant in breast MRI, where large numbers of quantitative imaging features may be generated relative to the number of available patients. Appropriate feature screening and selection are therefore important components of model development. Selection procedures should be prespecified when possible and incorporated within the training process to minimize overfitting and information leakage. Importantly, the identification of potentially informative MRI features does not by itself establish their predictive or clinical value. Robust internal validation and, when feasible, independent external validation are necessary to determine whether predictive performance and selected features are reproducible across patient populations and imaging settings. Thus, the clinical utility of machine-learning models depends not only on predictive performance, but also on methodological rigor, feature-selection procedures, interpretability, validation, and reproducibility.

So, despite the strong predictive performance demonstrated by radiomics-based models, clinical implementation will likely depend less on further incremental improvements in predictive performance than on the development of standardized imaging protocols, reproducible feature extraction methods, and externally validated workflows that can be implemented consistently across institutions. In addition, future predictive models should be evaluated not only by discrimination metrics such as AUC but also by calibration, and measures of clinical utility. as excellent statistical performance does not necessarily translate into improved patient outcomes. Well calibrated models are particularly important when predicted probabilities of response may inform individual patient management, while approaches such as decision curve analysis can help determine whether incorporating a model into clinical decision-making provides added benefit across clinically relevant decision thresholds.

Nevertheless, significant challenges remain before MRI-based predictive models can be routinely incorporated into clinical decision-making. Most studies were limited by relatively small sample sizes, retrospective or single-center design, heterogeneous MRI protocols, and variability in segmentation and feature extraction techniques. Across the included studies, sample sizes ranged from 25 to 259 patients, which may limit the statistical power and increase the risk of model overfitting, particularly for studies using machine-learning approaches with multiple imaging-derived predictors. Consequently, the high predictive performance reported in several individual studies should be interpreted cautiously, as estimates derived from small cohorts may be less stable and may not be reproduced in larger, more heterogenous populations. The recurrence of small cohorts across the literature therefore limits the strength and generalizability of conclusions regarding the comparative performance of MRI-based biomarkers. This concern is particularly relevant to radiomics-based models, in which many candidate imaging features may be evaluated relative to the number of patients and outcome events. In smaller cohorts, this high dimensionality increases the risk of overfitting, whereby a model may demonstrate strong performance in the development dataset but fail to maintain that performance in independent populations. Although external validation in several included studies strengthens the evidence for these approaches, validation cohorts were generally limited in size, and broader multicenter validation remains necessary. The I-SPY2 trial-derived cohorts evaluated by Li et al. and Liu et al. provide the advantage of multicenter data; however, trial-specific eligibility criteria and treatment protocols may differ from those encountered in routine clinical practice. Accordingly, the performance of imaging biomarkers derived from these cohorts may not fully generalize to broader, non-trial patient populations or alternative treatment regimens. Immunotherapy regimens also varied across the included studies, and the specific checkpoint inhibitor was not consistently reported. For example, Zou et al. described treatment with a PD-1 inhibitor without specifying the agent, limiting the ability to determine whether imaging findings are generalizable across specific immunotherapy regimens [37]. Given the small number of included studies (*n* = 10) and substantial clinical, methodological, and imaging heterogeneity across studies, quantitative meta-analysis was not performed, as pooling estimates was considered unlikely to yield a meaningful summary effect and could potentially result in misleading conclusions. Collectively, these limitations reduce the certainty and generalizability of current evidence. The findings should be interpreted cautiously given the small number of included studies (*n* = 10), small study populations, and heterogeneity in MRI methods and treatment protocols. These factors may contribute to overfitting and limit the generalizability of reported predictive performance. In addition, publication bias cannot be excluded, as studies reporting positive findings may be more likely to be published than those with negative or inconclusive results. Therefore, reported AUC values should be interpreted cautiously and require validation in larger, independent cohorts.

Ultimately, rather than replacing tissue-based biomarkers, MRI may be most valuable as a complementary tool that provides whole-tumor, longitudinal assessment of treatment response and immune dynamics. Tissue-based biomarkers such as PD-L1 expression, tumor mutational burden, and immune gene signatures provide molecular information from sampled tumor tissue, whereas MRI can noninvasively characterize spatial heterogeneity across the entire tumor and can be repeated throughout treatment. Integrating these complementary sources of information may therefore provide a more comprehensive assessment of tumor biology and treatment response than either imaging or tissue biomarkers alone. We acknowledge that the use of PubMED as a single database may potentially limit the comprehensiveness of this review. Additionally, our restriction of the search to English language publications may have introduced a potential risk of language bias.

Future research should prioritize prospective multicenter validation studies with standardized imaging acquisition and analysis pipelines. Integration of MRI biomarkers with genomic, transcriptomic, and immune profiling data may further improve predictive accuracy and could ultimately help identify patients most likely to benefit from treatment escalation or de-escalation strategies. For example, Han et.al [34] linked DCE-MRI radiomic features with transcriptomic characterization of the tumor microenvironment, illustrating how imaging and molecular data may be integrated to characterize immune phenotype and treatment response. However, the included studies did not prospectively evaluate MRI-guided treatment modification. Clinical implementation of MRI-based adaptive treatment strategies will require reproducible performance across institutions, integration into clinical workflows, consideration of regulatory requirements, and prospective interventional trials demonstrating that such strategies improve patient outcomes. Additionally, continued development of explainable AI and reproducible radiomics workflows will be essential for clinical translation.

## 5. Conclusions

Overall, the current evidence suggests that MRI—particularly when incorporating multiparametric imaging, diffusion-weighted techniques, and radiomics-based analysis, may have potential as a noninvasive tool for predicting immunotherapy response in breast cancer. However, the small and heterogenous evidence base limits conclusions regarding its clinical utility. Although the included studies suggest that MRI retains predictive value in patients receiving NACI, direct comparisons with NAC alone remain limited, and further prospective validation is needed. While MRI-based prediction of treatment response could potentially inform future treatment escalation or de-escalation strategies, the studies included in this review did not evaluate whether MRI-guided treatment adaptation improves patient outcomes. Prospective clinical trials demonstrating clinical benefit, along with standardized imaging and analysis protocols and consideration of practice and regulatory implementation challenges, are needed before MRI-based adaptive treatment strategies can be incorporated into routine clinical practice.

## Figures and Tables

**Table 1:** Summary of studies evaluating MRI-based prediction of pathological complete response (pCR) in breast cancer patients undergoing neoadjuvant chemoimmunotherapy. NACI = neoadjuvant chemoimmunotherapy, NAC = neoadjuvant chemotherapy, TNBC = triple-negative breast cancer; DCE = dynamic contrast-enhanced MRI; DWI = diffusion-weighted imaging; ADC = apparent diffusion coefficient; AUC = area under the curve; NR = not reported; FTV = functional tumor volume; HEV = High Enhancement Volume.

Author/Year/PMID	Country (Institution)	Study Type	Population (*n*)	Receptor Type	MRI Image Type	Model Type	Formal Predictive Model (Yes/No)	Predictor Variables Included in Model/Analysis	Outcome Variable	AUC (or Accuracy/Sensitivity/Specificity/Association)
Spriet et al., 2025 [35] (PMID: 40434685)	France (Oscar Lambret Center)	Mixed retrospective/prospective single-center study	Early-stage TNBC treated NACI; total *n* = 36, all treated with NACI	TNBC	T1/T2, DCE, DWI, UF-DCE	Diagnostic accuracy analysis	No	UF-MRI complete/partial response, full-protocol MRI complete/partial response	pCR	Sensitivity 0.78/Specificity 0.69
Li et al., 2022 [31] (PMID: 36139594)	USA (I-SPY 2 multicenter cohort)	Retrospective multicenter study (trial-derived cohort)	HER2- breast cancer treated with NACI; total *n* = 103, NACI *n* = 28, NAC alone *n* = 75	HR+/HR−; includes TNBC subset	T1/T2, DWI (apparent diffusion coefficient (ADE)), DCE	Multivariable logistic regression	Yes	Percent change in ADC metrics and FTV	pCR	AUC 0.73
Liu et al., 2025 [36] (PMID: 40736162)	China (Guangdong Provincial People’s Hospital; external validation: I-SPY 2)	Retrospective, single center with external validation	TNBC treated with NACI; total *n* = 119, training *n* = 90, external test *n* = 29	TNBC	T2, DCE	Multivariable logistic regression model	Yes	Tumor unifocality, early tumor shrinkage, percentage enhancement reduction; additional MRI morphology and enhancement features assessed	pCR	AUC 0.88
Ramtohul et al., 2024 [32] (PMID: 39225608)	France (Institut Curie, Paris and Saint-Cloud)	Prospective dual-center study	Early-stage TNBC treated with NACI; total *n* = 195, training *n* = 112, external test *n* = 83	TNBC	T2, DCE/perfusion	Radiomics model using LASSO feature selection + multivariable logistic regression nomogram	Yes	Radiomics features from K_trans_, V_e_, and Slope_max_ pharmacokinetic maps; combined nomogram included radiomics score, tumor grade, and Ki-67	pCR	AUC 0.86
Ramtohul et al., 2025 [26] (PMID: 40590696)	France (Institut Curie; Institut Godinot; Institut Oscar Lambret)	Prospective multicenter study	Stage II–III TNBC treated with KEYNOTE-522 NACI; total *n* = 259, training *n* = 175, external test *n* = 84	TNBC	T2, DCE	Multivariable logistic regression model + radiomics model (LASSO logistic regression)	Yes	Model 1: rCR (radiologic complete response), baseline nodal status, Ki-67 Model 2: Radiomic score + lesion count (in residual-enhancement/non-rCR cases)	pCR	Model 1: AUC = 0.88 Model 2: AUC = 0.80
Zou et al., 2024 [37] (PMID: 37985386)	China (Guangdong Provincial People’s Hospital)	Retrospective single-center study	TNBC treated with NAC or NACI; total *n* = 99, NAC *n* = 65, NACI *n* = 34	TNBC/HER2+	T2, DWI	Comparative statistical analysis	No	Tumor shrinkage pattern (concentric shrinkage, diffuse decrease, no change), treatment regimen (NAC vs. NACI)	pCR	CS vs. diffuse shrinkage: NAC 53% vs. 36%, NACI 20% vs. 68% (*p* = 0.004). pCR: NACI 82% vs. NAC 45% (*p* < 0.001). Within NACI, pCR was 17% of pts with CS vs. 56% of pts with diffuse decrease pattern (*p* = 0.903).
Han et al., 2024 [34] (PMID: 38287356)	China (Guangdong Provincial People’s Hospital)	Retrospective multicohort study with external validation	Breast cancer cohorts for radiomics-based tumor microenvironment and immunotherapy response prediction; discovery cohort *n* = 94, internal validation *n* = 24, immune phenotype cohort *n* = 158, immunotherapy cohort *n* = 77	TNBC	DCE	Random forest machine learning radiomics model	Yes	Intratumoral and peritumoral radiomics features from DCE-MRI	pCR/partial response vs. stable disease/progressive disease in a 6 month follow-up; tumor microenvironment phenotypes of “immune-inflamed cluster” vs. “immune-desert cluster”	AUC 0.784 (immunotherapy response prediction); AUC 0.814–0.855 (immune phenotype classification);
Dimaio et al., 2026 [21] (PMID: 41498827)	Brazil (Camargo Cancer Center)	Retrospective single-center observational diagnostic study	TNBC treated with NAC or NACI; total *n* = 111, NAC *n* = 59, NACI *n* = 52	TNBC	DCE, STIR	Diagnostic performance/ROC analysis	No	Intratumoral and peritumoral radiomics features from DCE-MRI	pCR	AUC 0.736
Weinfurter et al., 2025 [38] (PMID: 40945432)	USA (Moffitt Cancer Center)	Retrospective single-center study (trial-based cohort)	Stage II–III TNBC treated with investigational NACI (NAC + T-VEC); total *n* = 25, all received investigational NACI	TNBC	DCE, T2, ADC	Quantitative imaging biomarker analysis	No	rCR, lesion morphology, enhancement kinetics	pCR	pCR: 48%. Higher %FTV (84.8% vs. 67.4%, *p* = 0.022) and %HEV (93.3% vs. 81.3%, *p* = 0.010) were associated with pCR. Above-average %FTV/%HEV predicted higher pCR (71% vs. 18%, *p* = 0.008; 69% vs. 11%, *p* = 0.006) and lower tumor cellularity (*p* = 0.023, 0.003).
Javed et al., 2026 [39] (PMID: 41913651)	UK (University College London Hospital)	Retrospective single-center study	Early breast cancer treated with NAC/NACI, TNBC subgroup stratified by immunotherapy use; total *n* = 201, HER2+ *n* = 83, TNBC *n* = 64, ER+/HER2− *n* = 54	TNBC	DCE, DWI	Statistical analysis	No	FTV, HEV, voxel-based temporal enhancement habitat metrics	pCR	False-negative rate of MRI (MRI showed complete response but path showed residual cancer): 21/175 (12%) for pCR in axilla and 15/175 (8.5%) for pCR in breast. Overall false-negative rate for MRI (MRI showed complete response, but path showed residual cancer) for pCR was 28/175 (16%). Overall false-positive rate of MRI (MRI showed residual tumor, but path showed no residual cancer) for pCR was 2/175 (1.1%). False negative (MRI showed no tumor, but path showed residual tumor) was 6.8% for HER2+, 15.3% for TNBC, and 37.2% for ER+

## Data Availability

No new data were created or analyzed in this study. Data sharing is not applicable to this article.

## Abbreviations

The following abbreviations are used in this manuscript:

NAC	Neoadjuvant chemotherapy
TNBC	Triple-negative breast cancer
ER	Estrogen receptor
PR	Progesterone receptor
HER-2	Human epidermal growth factor receptor-2
ICI	Immune checkpoint inhibitor
PD-l	Programmed cell death protein 1
PD-L1	Programmed cell death ligand 1
NACI	Neoadjuvant chemoimmunotherapy
pCR	Pathologic complete response
MRI	Magnetic resonance imaging
DCE-MRI	Dynamic contrast-enhanced MRI
DWI	Diffusion-weighted imaging
ADC	Apparent diffusion coefficient
rCR	Radiologic complete response
UF-MRI	Ultrafast MRI
FTV	Functional tumor volume
HEV	High enhancement volume
TME	Tumor microenvironment
T-VEC	Talimogene laherparepvec
